# Hepatoblastoma with solid and multicystic aspect mimicking a mesenchymal hamartoma: imaging and anatomopathologic findings

**DOI:** 10.1590/0100-3984.2015.0163

**Published:** 2017

**Authors:** Pedro Vinícius Staziaki, Bernardo Corrêa de Almeida Teixeira, Bruno Mauricio Pedrazzani, Elizabeth Schneider Gugelmin, Mauricio Zapparolli

**Affiliations:** 1 Hospital de Clínicas da Universidade Federal do Paraná (UFPR), Curitiba, PR, Brazil.; 2 Hospital Pequeno Príncipe, Curitiba, PR, Brazil.

**Figure 1:**
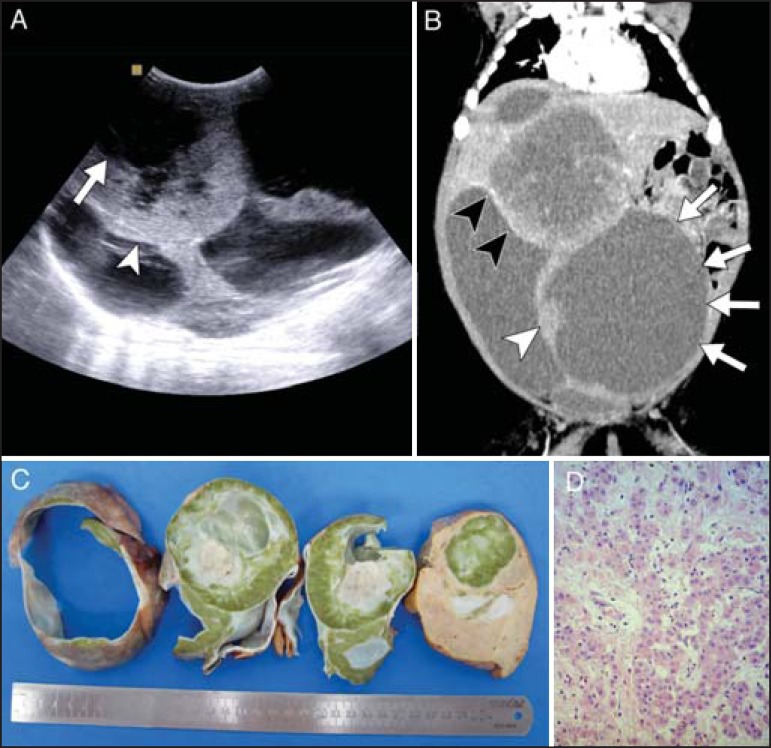
**A:** Ultrasound demonstrating a large heterogeneous mass comprising multiple cystic areas (arrow) and solid areas (arrowhead). **B:** Coronal computed tomography reconstruction during the arterial phase of contrast enhancement showing clearly delimited multiple cystic components (arrows) and solid components (white arrowhead) with heterogeneous and predominantly peripheral accumulation of contrast (black arrowheads). **C:** Photograph of the surgical specimen, showing components of cystic degeneration and central necrosis with adjacent pseudocysts. The emerald color is caused by biliary stasis inside the tumor. **D:** Histopathological section showing a mixed epithelial (fetal) pattern and mesenchymal hepatoblastoma (original magnification, ×100).

Dear Editor,

A 29-day-old infant was evaluated at our center for a hepatic mass found during gestation. Ultrasound revealed a heterogeneous lesion which comprised three anechoic areas with hypoechoic debris and a predominantly hyperechoic central region with signs of vascularization on Doppler imaging (Figure 1A). Computed tomography showed a large bulging mass with three clearly defined cystic components with heterogeneous contrast enhancement of peripheral solid nodules (Figure 1B). The cystic component suggested a diagnosis of mesenchymal hamartoma. However, a highly elevated level of alpha-fetoprotein led to the diagnosis of hepatoblastoma, which tends to present as a solid lesion. An ultrasound-guided biopsy confirmed a mixed epithelial/mesenchymal hepatoblastoma (Figures 1C and 1D).

After the definitive diagnosis was made, the patient underwent chemotherapy with cisplatin and doxorubicin. Upon completion of the second cycle of chemotherapy, it was decided that the patient should undergo liver transplantation, which occurred at 8 months and 3 days of age. The surgical specimen showed large masses of an emerald color with components of cystic degeneration and central necrosis with adjacent pseudocysts. The histopathological study confirmed a mixed mesenchymal/epithelial hepatoblastoma throughout the neoplastic tissue, with formation of pseudocysts.

Liver tumors are not uncommon in adults^(1-4)^. Hepatoblastoma is the most common primary hepatic malignancy in children, accounting for nearly 80% of all malignant liver tumors^(5)^. Hepatoblastoma usually presents as an incidental finding of an asymptomatic abdominal mass in children under 5 years of age. On ultrasound, hepatoblastomas appear as predominantly solid masses that are hyperechoic relative to the adjacent liver, although hypoechoic fibrotic septa can also be seen. Epithelial hepatoblastomas may appear homogeneous, whereas mixed epithelial/mesenchymal tumors are heterogeneous (due to osteoid, cartilaginous, and fibrous components) and frequently contain echogenic calcifications with acoustic shadowing and anechoic foci representing hemorrhage or necrosis^(6)^. The appearance of hepatoblastoma on computed tomography is that of a well-defined mass with regular borders that is hypoattenuating in comparison with the adjacent hepatic parenchyma. The tumor commonly displays diffuse heterogeneous contrast enhancement. Approximately half of all hepatoblastomas appear lobulated or septated, especially on contrast-enhanced images^(6,7)^.

In the case presented here, the imaging findings indicated a different entity. The predominantly cystic appearance of the tumor was consistent with a cystic liver tumor, namely mesenchymal hamartoma. Mesenchymal hamartomas, which typically occur in children under 2 years of age, present as a large solitary neoplasm with variable amounts of solid and cystic components on ultrasound or computed tomography^(8,9)^. Our conclusion is that we should be aware of this rare mostly cystic presentation of hepatoblastoma, should we encounter cystic hepatic lesions in children under 3 years of age with elevated alpha-fetoprotein levels.
